# Efficacy and Safety of a Novel Submucosal Injection Solution for Resection of Gastrointestinal Lesions

**DOI:** 10.3390/jcm9041162

**Published:** 2020-04-18

**Authors:** Cristina Moles-Aranda, Raquel González-Pérez, Francisco Javier Gallego-Rojo, Olga Martínez-Augustin, Beatriz Clares-Naveros, Fermín Sánchez de Medina, José Antonio Morales-Molina

**Affiliations:** 1 Department of Pharmacy and Pharmaceutical Technology, School of Pharmacy, University of Granada, Campus Universitario de Cartuja, 18071 Granada, Spain; cmolesaranda@hotmail.com (C.M.-A.); beatrizclares@ugr.es (B.C.-N.); 2 Department of Pharmacology, CIBERehd, Instituto de Investigación Biosanitaria ibs.GRANADA, School of Pharmacy, University of Granada, Campus Universitario de Cartuja, 18071 Granada, Spain; raquel.gonzalez@ciberehd.org; 3 Instituto de Investigación Biosanitaria ibs.GRANADA, University of Granada, 18071 Granada, Spain; omartine@ugr.es (O.M.-A.); jomomo01@gmail.com (J.A.M.-M.); 4 Department of Digestive, Hospital de Poniente, Ctra. Almerimar, 04700 El Ejido, Almería, Spain; fgallegorojo@gmail.com; 5 Department of Biochemical and Molecular Biology II, CIBERehd, Instituto de Investigación Biosanitaria ibs.GRANADA, School of Pharmacy, University of Granada, Campus Universitario de Cartuja, 18071 Granada, Spain; 6 Nanoscience and Nanotechnology Institute (IN2UB), University of Barcelona, 08193 Barcelona, Spain; 7 Department of Pharmacy, H.U. Torrecárdenas, C/ Hermandad de Donantes de Sangre, 04009 Almería, Spain

**Keywords:** efficacy, safety, endoscopic submucosal dissection, endoscopic mucosal resection, gastrointestinal endoscopy, submucosal injection

## Abstract

Endoscopic mucosal resection (EMR) and endoscopic submucosal dissection (ESD) are minimally invasive and efficient techniques for the removal of gastrointestinal (GI) mucosal polyps. In both techniques, submucosal injection solutions are necessary for complete effectiveness and safety during the intervention to be obtained. The main objective of this study was to evaluate the efficacy and safety of a new sterile submucosal injection solution for EMR/ESD used within a clinical protocol in patients with intestinal polyps. We carried out a prospective study between 2016 and 2017 with patients who attended the Endoscopy Consultation—Digestive Department of Primary Hospital. Patients were selected for EMR/ESD after the application of clinical protocols. Thirty-six patients were selected (≥ 66 years with comorbidities and risk factors). Lesions were located mainly in the colon. Our solution presented an intestinal lift ≥ 60 min in EMR/ESD and a high expansion of tissue, optimum viscosity, and subsequent complete resorption. The genes *S100A9* and *TP53* presented an expression increase in the distal regions. *TP53* and *PCNA* were the only genes whose expression was increased in polyp specimens vs. the surrounding tissue at the mRNA level. In EMR/ESD, our solution presented a prolonged effect at the intestinal level during all times of the intervention. Thus, our solution seems be an effective and safe alternative in cases of flat lesions in both techniques.

## 1. Introduction

Nowadays, endoscopic mucosal resection (EMR) and endoscopic submucosal dissection (ESD) are the two most used techniques to remove gastrointestinal (GI) mucosal polyps. Both of them are minimally invasive techniques, efficient for the treatment of these polyps [1,2]. EMR enables a complete removal of suspect premalignant lesions with an efficacy greater than 90% [3]. This technique is especially useful in lesions < 15–20 mm that present low risk of metastasis. In bigger lesions, which are resected in a piecemeal fashion (pEMR), ESD is more useful [4]. This technique allows in bloc resection of superficial lesions, providing better histopathological diagnosis and decreasing the local recurrence rates [5,6,7]. In both techniques, submucosal injection solutions are used to delimit the area to be excised and to separate the lesion from the muscularis propria. This allows complete resection of the lesion, and avoids perforation risks, bleeding, and injury to the GI wall, thus favoring the rapid recovery of the patient [8,9]. So, the use of submucosal injection solutions is essential to ensure the effectiveness and safety of the intervention.

In Europe, there is a limited availability of commercial submucosal injections for EMR and ESD, so a number of different in-house solutions are used, with saline solution being one of them. However, the use of saline solution is associated with higher rates of residual lesions when compared with more viscous solutions [10]. The latter present the advantage of a longer-lasting effect. Some of these include dextrose or colloids like dextran, fibrinogen, autologous blood [11], gelofusine [12], or sodium hyaluronate [13]. Solutions containing hypromellose or glycerol might also be considered [13].

The lack of a standard commercial submucosal injection for EMR and ESD makes it necessary for hospitals to prepare their own solutions. Problems associated with this fact are the difficulty to prepare them, the lack of consistency of the injected solution, uncertain stability, and possible toxicity-associated problems. In addition, because of their non-optimal viscosity, the administration procedure can be a problem and, due to the short duration of the lesion elevation effect, repeated injections may be needed [14,15,16,17].

Taking into account all of these shortcomings, it is obvious that there is a need for a commercial optimal submucosal injection solution that may be easily prepared and administered, keeping a consistent composition. This solution should have an optimum viscosity to facilitate the injection and to elevate the lesion for a long time, avoiding repeated injections. To ease the physician endoscopist’s work, it should also facilitate the visualization of the edges of the lesion and the lesion itself. In addition, total resorption of the solution together with the absence of toxicity and side effects (tissue damage, bleeding, inflammation), and a physical-chemical and biological stability, is desirable [18,19]. Finally, a low cost would be advisable.

In our hospital, physician endoscopists have used a number of different solutions, such as 5% glucose solution, 10% glycerol solution, and gelofusine solution, in the last few years. As the results obtained were not completely satisfactory due to the previously mentioned problems, a review of the scientific literature was carried out in order to evaluate the safety and effectiveness of different solutions used in endoscopic submucosal dissection and endoscopic mucosal resection (Pubmed 19662019—MeSH: endoscopy submucosal dissection, endoscopy mucosal resection, submucosal injection solution, efficacy, safety). Initially, our idea was to design a suitable combination of substances to formulate the working solution, on the basis that this approach would produce better results than any individual ingredient separately. One additional advantage is the use of reduced concentrations of each active agent in the mixture, thereby minimizing the risk of adverse reactions. The pharmacy and digestive departments of our hospital in collaboration with a researcher from the School of Pharmacy of the University of Granada elaborated a series of analogues prioritizing efficacy, safety, and stability, until the formula was optimized to yield a solution that was since included in the clinical protocol approved in the hospital. At present, the research protocol is that all patients receive the new solution in both EMR and ESD. The main objective of this study was to evaluate the efficacy and safety of a new solution for EMR and ESD used within a clinical protocol in patients with intestinal polyps.

In addition to testing the effectiveness and security of this solution, we tested the hypothesis that studying the gene expression of inflammatory, cancerous, and proliferation markers in polyps and adjacent tissue could be useful to asset the efficiency of EMR and ESD procedures.

## 2. Materials and Methods

### 2.1. Primary Outcomes

To evaluate the efficacy of the new solution for EMR and ESD that was used within a clinical protocol in patients with intestinal polyps, we evaluated the polyp lift/intervention time ratio. To assess the safety, we evaluated adverse effects related to the submucosal injection solution.

### 2.2. Injection Solution

**Figure 1:**
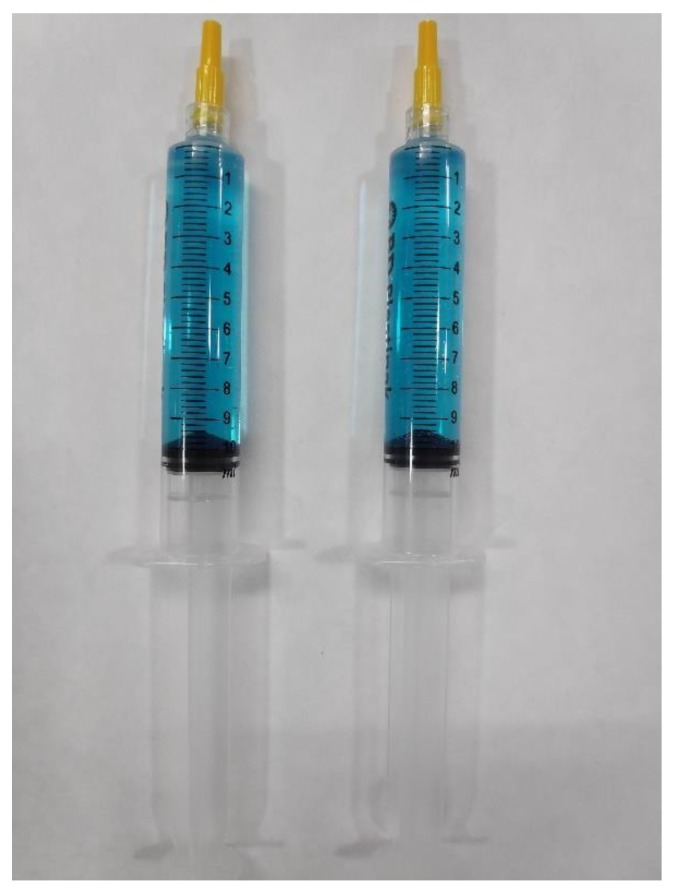
Representative picture of the submucosal injection solution in the syringe.

The EMR/ESD novel injection solution was designed based on scientific evidence and the following criteria: (1) Efficacy and safety reported for each one of the components; (2) potential benefits of the combination of all components in a single solution; (3) the viscosity to obtain a stable effect; and (4) the addition of a dye to facilitate submucosal lesion visualization (assessment of the lesion depth and location of the edges). The final product was patented as a sterile injectable solution (P20163000; ES 2621877 A1; OEPM). The solution was prepared in sterile conditions (Horizontal Laminar Flow Cabinet—Class 100) and subjected to strict physicochemical and microbiological characterization and control. Physicochemical stability conditions were considered acceptable with pH = 5.0–6.5, relative density 1.05–1.09 g/mL, infrared spectrum with a correlation coefficient ≥ 0.95, viscosity 20–50 mPas, and correct visual analysis of solution (see Figure 1 showing the solution with methylene blue). A sterility test, endotoxin assay (<0.5 EU/mL), and microbiological assay (chromogenic technique—European Pharmacopoeia IX Ed. (2.6.14-D method)) were also performed.

### 2.3. Prospective Study

We carried out a prospective study from January 2016 through to December 2017 with patients who attended the Endoscopy Consultation—Digestive Department of Primary Hospital. Patients were selected for EMR or ESD after the application of clinical protocols. The inclusion criteria were: Patients diagnosed of polyps on the intestinal submucosal. Patients were cited for resection of lesions in < 3 months from diagnosis. Exclusion criteria included a documented allergy to any of the submucosal injection solution components; uncorrected coagulation disorders, with an INR equal to or greater than 1.5, or being under active treatment with antiplatelet drugs; lack of signed informed consent, non-acceptance, or contraindication of surgical or anesthetic techniques; and difficulty in understanding, by the patient, the conditions of the application of the clinical protocol. The withdrawal criteria included the following: Patients were withdrawn from the study according to the criteria established in Clinical Protocol—Hospital de Poniente. v.1. 01/01/2015, when the patient presented a moderate or severe adverse reaction to components of the solution or upon the withdrawal of consent. 

For both procedures, high-definition colonoscopes (HD) and optical magnification were used, as well as a water jet system incorporated in the endoscope through a low-flow pump system (‘water jet’ system). CO_2_ infusers were also used to perform endoscopies of long duration. The polypectomy snares used had an oval and multifilament morphology with sizes of 30 and 10 mm, facing large lesions in Piecemeal. The monopolar current with a coagulation mixture was used according to the electrosurgical unit manufacturer’s recommendations. All interventions were recorded in HD format—Medicap® USB 300 (Medicapture Inc, Plymouth Meeting, PA, USA)—after giving the patients the corresponding information sheet and obtaining their signed informed consent. This study was approved by the Committee of Clinical Trials with Medicines.

According to the Clinical Protocol of the Digestive Department, prior to resection of a precocious neoplastic flat lesion, a comprehensive examination with electronic chromoendoscopy (FICE system of Fujifilm) and/or chromoendoscopy with methylene blue or indigo carmine to morphologically typify the lesion according to the Paris–Japan classification was performed [20]. In addition, the crypt pattern of the lesion was typified according to the Kudo classification [21]. It is not recommended to take biopsies at this point of the study because this can interfere with the resection technique and it does not contribute to the classification of the lesions or the treatment. These data, in a first colonoscopy, allow the physician to assess the possibility of completely resecting the lesion with either endoscopy (EMR or ESD) or surgical treatment (transanal endoscopic microsurgery (TEMS) and variants for low rectal lesions, laparoscopic in other locations, or even open surgery) [21]. According to the study protocol, the new solution described above was used for EMR and ESD in all patients, except in the case of allergy or intolerance, where a standard solution of 0.9% saline serum was used. No cases of allergy or intolerance were observed in our study.

### 2.4. EMR/ESD Procedure

The solution was injected into the intestinal submucosal using a digestive endoscope with a 240-cm flexible probe. This device has a luer-lock syringe coupled at its proximal end containing the mucosectomy solution. A 23-gauge endoscopic injection needle is attached at the distal end. When performing the resections, the following parameters were recorded: Number and size (cm) of excised polyps, number of resections for complete removal of lesions (sessions), solution injections per polyp (number), polyp reinjections (number), injected volume in each lesion (mL), polyp lift (min), intervention time (min), polyp lift/intervention time ratio, time to resorption/intervention time ratio, vascularization of tissue (Good/Fair/Bad), bleeding during intervention (YES/NO), type of bleeding (MILD/MODERATE/SEVERE), complications of intervention, and evolution of tissue after administration of the drug (inflammation/tissue damage). After histological analysis, polyps were classified as low-grade dysplasia or high-grade dysplasia.

For histological analysis, a sample of polyp was placed in 10% formaldehyde for pathological analysis. In addition, for RT-qPCR analysis and to characterize the quality of the extracted tissue, biopsies of polyps and adjacent tissue were obtained (first 1 sample of polyp and after 1 sample of adjacent tissue). Adjacent tissue biopsies were obtained 1–2 cm away from the polyp. These samples were collected in RNase-free tubes containing RNAlater. For gene expression analysis, total RNA was obtained with Trizol^®^ reagent using the method described by the manufacturer (Invitrogen, Barcelona, Spain), and the quantity and integrity of RNA were assessed by spectrophotometry (260/280 nm absorbance ratio). Here, 1 µg of RNA was retrotranscribed (iScript, BioRad, Alcobendas, Spain) and the following markers were determined by qPCR: Cyclooxygenase 2 (COX2), intestinal alkaline phosphatase (ALPI), the calprotectin subunits S100A8 and S100A9, and myeloperoxidase (MPO) as inflammatory markers; and proliferating cell nuclear antigen (PCNA), tumor protein 53 (TP53), carcinoembryonic antigen gene family (CEA), prominin 1 (PROM1), and Ki-67 (MKI67) as proliferation and cancerous markers. The following primers were used: GGA GAA AAG GAA ATG TCT GC / GTA GGC AGG AGA ACA TAT AAC (COX2), ATC TCA TGG GCC TCT TTG / GCC TCT GTC ATC TCC ATC (ALPI), GTA TAT CAG GAA AAA GGG TGC / TAC TCT TTG TGG CTT TCT TC (S100A8), GCA AAA TTT TCT CAA GAA GGA G / CCA TCA GCA TGA TGA ACT C (S100A9), CTG TGT AGT AAA GAT GCC TTC / TCT CTA TGG TAA CAG CTT CC (PCNA), CCA TGG AAC TCC TAT CCT AC / TTG ACT TGG ACA ACA CAT TC (MPO), ACC TAT GGA AAC TAC TTC CTG / ACC ATT GTT CAA TAT CGT CC (TP53), CTT CAT TTC AGG AAG ACT GAC / TTA GTA GAG ATG GGG TTT CAC (CEA), AAG CAT TGG CAT CTT CTA TG / TTT GCT CTG GAG TTT CAT TC (PROM1), GAC AGA GGT TCC TAA GAG AG / AAC AAT CAG ATT TGC TTC CG (MKI67). Hypoxanthine guanine phosphoribosyl-transferase (*HPRT*), peptidylprolyl Isomerase B (*PPIB*), and 18Sribosomal RNA (18S) were used as reference genes.

### 2.5. Follow-Up of Patients after EMR/ESD

All patients were evaluated in the area of patient recovery at the digestive department—endoscopy unit for a period of about 120 min to detect early complications (abdominal pain, rectal bleeding, fever). When no secondary events were detected, patients were discharged with a copy of the report and recommendations about possible late complications. In complex cases, patients were admitted for 24–48 h to the digestive department. Subsequently, they were cited after one month at the endoscopy consultation to assess the histology of lesion. Subsequently, an endoscopic follow-up was performed at 3, 6, and 12 months. Further controls were carried out every 1, 2, 3, or 5 years based on other factors (patient’s age, family history of colorectal cancer and polyps).

### 2.6. Statistical Analysis

Statistical analysis of real-time PCR data was performed using Student’s *t*-test when two groups were compared, or one-way analysis of variance followed by least significant difference post-hoc tests for 3 group comparisons. Pearson’s correlation test was applied to measure the statistical relationship between two variables. Differences were considered significant at *p* < 0.05.

## 3. Results

### 3.1. Clinical Study: Efficacy and Safety

**Figure 2:**
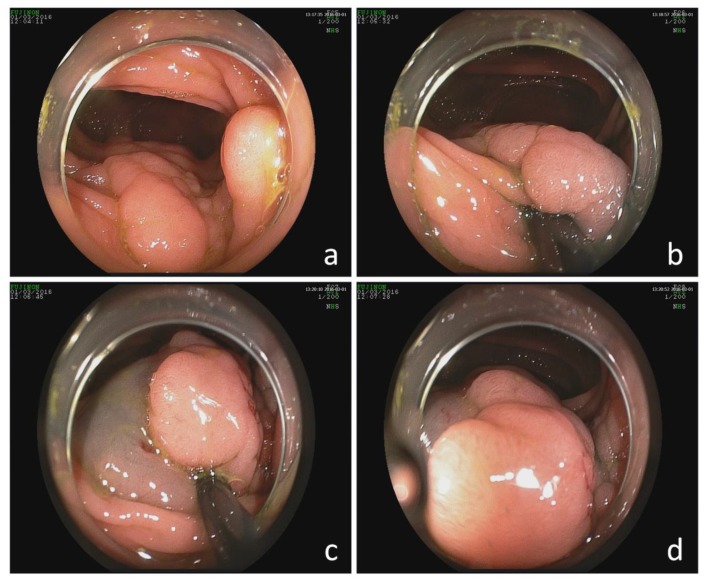
Endoscopic image of the colon in a patient intervened by endoscopic mucosal resection (EMR). (**a**) Endoscopic image of the colon. (**b**) Endoscopic image after intestinal submucosal injection. (**c**) Endoscopic image of the initial lift of a polyp in the intestinal mucosal after submucosal injection. (**d**) Endoscopic image of the final lift of a polyp in intestinal mucosal after submucosal injection.

Thirty-six patients were selected. Lesions were located in the colon, rectum, or caecum in 26, 4, and 6 patients, respectively. Table 1 shows the demographic features and clinical data of the patients. The characteristics of the lesions are described in Table 2. Data pertaining to the execution of the resection technique and complications of the intervention are provided in Table 3. While the same injection solution was used in all patients, 50% of them were applied EMR and the other 50% ESD, as the doctors’ learning curve evolved. A representative endoscopy (EMR) is shown in Figure 2.

Despite the longer intervention time in ESD vs. EMR, in all cases lasting more than 60 min, we observed a maintained lift of polyps with effective separation of the intestinal mucosa from the area supplied by blood vessels. In addition, we had to re-inject 3 polyps in EMR and 2 polyps of ESD out of 36 patients (13.9%) due to perforation of the intestinal mucosal layer during the intervention and the consequent extravasation of the solution. No patient presented a loss of submucosal elevation that impeded the procedure and one reinjection was sufficient in most cases, although three patients in EMR and two patients in ESD required re-injection. Of note, in the EMR group, five patients needed a new session for complete excision of their polyps vs. two patients in the ESD group.

Patients with a history of cancer and/or a family history of CRC were prioritized to ESD (66.7 vs. 21.4) because of the characteristics of the lesions.

After clinical follow-up, patients did not generally show significant complications associated with the intervention (i.e., other than bleeding). In a few cases, adrenaline was used to reduce the potential risk of hemorrhage and to promote a faster recovery. Subsequently, the solution injected into the submucosa was reabsorbed without problems. 

Prior to the intervention, all patients presented a good tissue vascularization in the polyp area. In all patients, the revascularization of tissue submitted to the intervention was complete. We only observed massive bleeding in one patient (ESD group). Bleeding was controlled with epinephrine injections and endoscopic hemoclips. We did not observe any additional complications in our patients during the intervention. In reviews of the patients after the intervention, in all cases, we observed a complete resorption of the submucosal injected solution and the absence of adverse effects potentially associated with its use.

### 3.2. Results of the Expression of Markers by PCR

**Figure 3:**
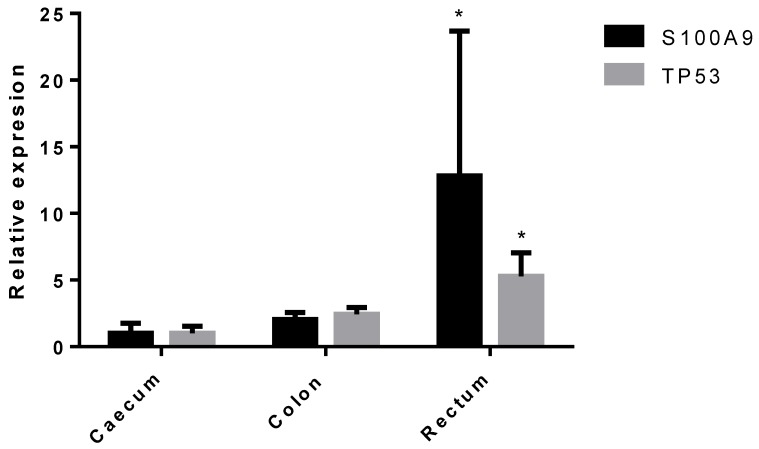
*S100A9* and *TP53* expression by tissue location. Data are expressed as 2^−ddCt^. * *p* < 0.05 vs. caecum.

All the genes analyzed were expressed at comparable levels in the polyp samples obtained from the colon, rectum, and caecum, except *S100A9* and *TP53*, whose expression was found to be increased in the distal regions: Rectum (*p* = 0.00874 and *p* = 0.005, respectively) and colon (*p* = 0.0233 and *p* = 0.0339, respectively) versus caecum (Figure 3).

**Figure 4:**
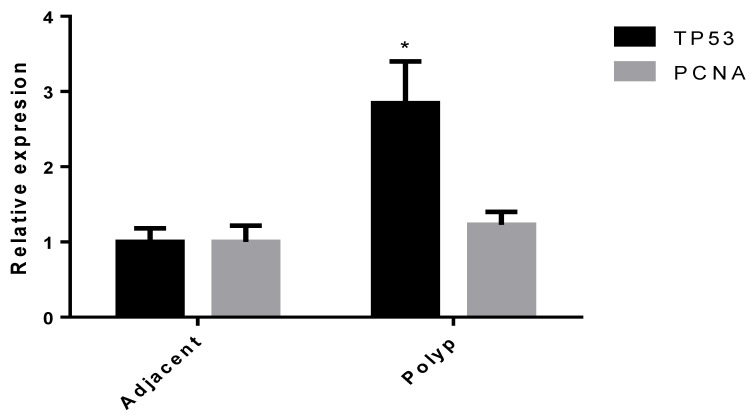
*TP53* and *PCNA* expression in polyp vs. adjacent tissue. Data are expressed as 2^−ddCt^. * *p* < 0.05 vs. adjacent.

**Figure 5:**
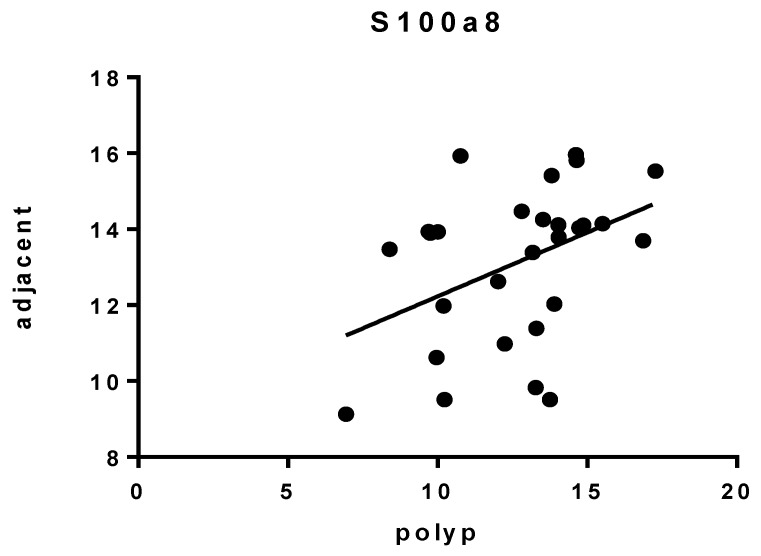
*S100A8* correlation expression between polyp and adjacent tissue. Data are expressed as ΔCt.

Regarding the expression of selected genes in polyp vs. adjacent tissue, the results obtained show few differences. Our data indicate that *TP53* and *PCNA* were the only genes whose expression was increased in the polyp specimen at the mRNA level (*p* = 0.0428 and *p* = 0.0625, respectively) (Figure 4). As a rule, there was no correlation in the expression of the genes examined between these two sites, with the sole exception of *S100A8*, which exhibited a weak but significant correlation (*r* = 0.4148, *p* = 0.0282) (Figure 5).

**Figure 6:**
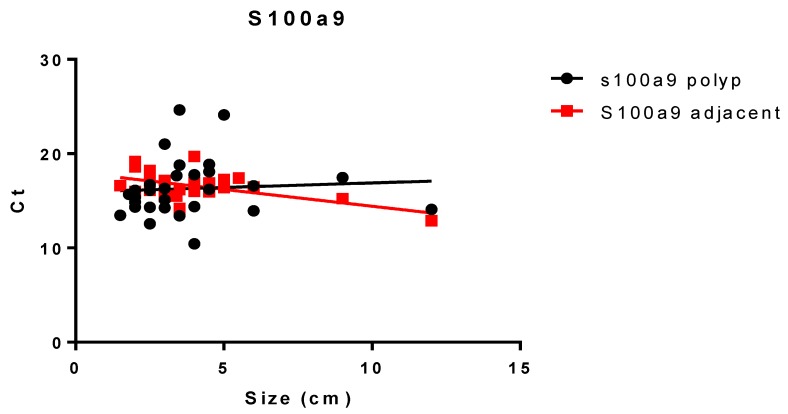
*S100A9* correlation expression in polyp or adjacent tissue vs. size of lesion. Data are expressed as ΔCt.

When the correlation between the clinical data and gene expression was studied, a positive correlation between the expression of *S100A9* and the size of the lesion in adjacent but not polyp tissue was found (*p* = 0.0043, *r* = −0.515) (Figure 6).

## 4. Discussion

The use of an appropriate solution for EMR and ESD is key for the optimal application of these techniques. In the United States and Japan, Sigmavisc^®^ (Hyaltech Ltd., Livingston, UK) and Muco-Up^®^ (Johnson and Johnson, Tokyo, Japan), two solutions of hyaluronic acid with an indication profile similar to that of our solution, have been commercialized. In Europe, Eleview^™^ and Orise have been approved. Nevertheless, their high cost and availability problems may limit their use [22]. In addition, the ideal composition for this type of solution has not yet been standardized. In this article, we described the results obtained using a new solution that, when injected in the intestinal submucosa, produces a long-lasting elevation of lesions, separating the polyp from the area irrigated by blood vessels for more than 60 min. In our study, we only had to re-inject 5 out of 36 patients (13.9%) due to perforation of the mucosal layer during the intervention, with the consequent extravasation of the solution. We did not observe resorption of the solution before the end of the intervention in any patient. A long-lasting lift is especially important for ESD, a procedure that can last several hours [23]. The exceptional duration of the effect of our solution, avoiding the need for additional injections, contrasts with that of other solutions, such as the recently commercialized Eleview^™^ (Cosmo pharmaceuticals NV, Dublin, Ireland), with a lifting time of only 12.5 min. Other alternatives, such as saline serum and glycerol solutions, are cheap and easy to prepare, but they also have the limitation of a short lifting time. In addition, saline serum is quickly absorbed into the adjacent mucosa, resulting in a flattening of the submucosal elevation, which makes repeated injections necessary. On the other hand, solutions containing glycerol are highly viscous, hindering their administration into the submucosa. An additional problem of glycerol-containing solutions, like the commercially available Glyceol^®^ (marketed in Japan, with other indications [8,14,24]), is the production of ‘fumes’ that hamper the END and ESD procedures being performed [25]. Our solution avoids both high viscosity and the production of fumes, thus allowing for easy administration and endoscopist performance.

Regarding safety and side effects, our data indicate that the new solution is associated with a low risk of hemorrhage and quick recovery. After the intervention, the injected solution was reabsorbed completely, without producing adverse effects during follow up. Some authors have reported that the risk of adverse effects is mainly associated with the procedural technique [26]. 

In our study, we evaluated the expression of inflammatory, proliferation, and cancerous markers in both polyps and adjacent tissue. This approach was taken chiefly to assess the occurrence of possible differences between the excised samples and the surrounding tissue, which might be expected on the basis of histology. In general, we found only slight differences in marker expression between polyps and adjacent tissue, with essentially no correlation. *S100A8* was the only exception, but even then, the correlation was weak, albeit significant. This, together with the fact that *S100A9* was not differentially expressed in polyp vs. adjacent tissue, suggests that the area surrounding the polyp is phenotypically unrelated. In other words, this is consistent with adequate polyp excision. On the other hand, *S100A9* expression in adjacent tissue was positively correlated to the extension of the lesion, which is suggestive of an augmented inflammatory response in relatively large polyps. Both S100A8 and S100A9 are members of the S100 family of calcium-binding proteins that play roles in the innate immune system. They can exist as monomers or as a heterodimer (i.e., calprotectin). Overexpression of these proteins has been observed in inflammation as well as in tumor cells and is associated with poor cell differentiation in cancers of glandular cell origin [27]. It has been reported that the expression of S100A8 or S100A9 in stromal cells of CRC is associated with larger-sized tumors [14]. Nevertheless, little research has been done on their expression in tumor cells of CRC and their correlation with CRC progression.

Only the expression of *TP53* was found to be significantly increased in polyps vs. adjacent tissue. *TP53* encodes tumor protein p53, a long-established tumor suppressor protein. Although p53 expression may be downregulated in colonic cancer, increased expression has been documented in relation to the progression of colorectal adenomas [28] and with high-grade dysplasia in adenomas [29,30]. In turn, no changes were detected for *PCNA*, *PTGS2* (COX2), *CEACAM5* (carcinoembryonic antigen cell adhesion molecule 5), or *MKI67* (marker of proliferation Ki67). Thus, p53 emerges as a relevant marker in this context. However, it should be noted that no correlation with malignancy was noted in any case. 

## 5. Conclusions

Our solution allowed full effectiveness and high safety in all cases of EMR and ESD. Advantages of this solution are a prolonged effect at the intestinal level, high expansion of tissue by injecting only a small amount of product, optimum viscosity, and complete resorption. One of the strengths of this pilot study lies in it being performed by a single, experienced endoscopist. Its main weakness is the small number of patients studied. Thus, these preliminary results should be accompanied by comparative clinical studies between the different commercial solutions, guaranteeing the absence of possible secondary effects derived from their use. 

## 6. Patents

In relation with this project, a sterile injectable solution was patented (P20163000; ES 2621877 A1; OEPM).

## Figures and Tables

**Table 1:** Demographic and clinical data (mean).

	**EMR**	**ESD**
Patients	72.2% men (13/18)	77.8% men (14/18)
Age (years)	69.2 (range: 37–86)	66.0 (range: 36–85)
Previous polypectomy	0.33 (range: 0–2)	0.75 (range: 0–5)
Lesion site (%)	77.8% in colon (14/18)	66.7% in colon (12/18)
**Comorbidities**		
Dyslipemia (%)	48.5	64.7
Cardiovascular Pathology (%)	34.2	17.7
Hypertension (%)	69.9	70.6
Diabetes mellitus (%)	50.0	41.2
Chronic obstructive pulmonary disease (%)	21.4	17.6
Smoker (%)	50.0	45.4
Enolism (%)	42.9	12.5
Hemorrhoids (%)	70.0	76.5
Background other type of cancer (%) / family history of CCR (%)	21.4	66.7

EMR: endoscopic mucosal resection; ESD: endoscopic submucosal dissection; CCR: Colorectal Cancer.

**Table 2:** Characteristics of lesions.

Patient Code	Lesions (*n*)	Paris Classification	Size of Lesion (cm)
2	2	0-IIa/0-IIc/IIa	2.2/1.2
3	1	0-Is/IIa	3.5
4	1	0-IIa	2
5	1	0-Is	4
6	1	0-Is	5
7	1	0-IIa	1.5
8	2	0-IIa/0-IIb	2/2.5
9	1	0-Is/IIa	3.5
10	2	0-IIa/0-Is	1/2.5
11	1	0-Is	4
12	1	0-IIa	2.2
13	1	0-IIa	2.4
14	1	0-IIs	2.5
15	1	0-IIa	1.5
16	1	0-IIb	2.5
17	1	0-IIa	2.4
18	2	0-Is/0-IIa	4/1.5
19	1	0-Is	4
20	1	0-Is	12
21	1	0-Is	3
22	1	0-IIc/IIa	1.8
23	1	0-IIb	4.5
24	1	0-Is	6
25	1	0-IIa	2
26	1	0-IIa	3
27	1	0-Is/IIa	9
28	1	IIa+IIb	3
29	1	0-IIa	4.5
30	1	0-IIc/IIa	2
31	1	0-Is/IIa	3
32	1	0-IIa	2
33	1	0-Is/IIa	6
35	1	0-IIa	2
36	1	0-Is/IIa	4.5
38	1	0-Is	3
39	1	0-Is	3.5

**Table 3:** Resection technique data and complications of intervention (mean).

**Resection Technique Data**	**EMR**	**ESD**
Malignancy of tissue (%)	27.8 (5/18)	23.5 (4/17)
Excised polyps (*n*)	2.9 (range: 1.5–4)	3.6 (range: 2–9)
Resections for complete removal of lesions (*n*)	5.5 (range: 4–10)	2.1 (range: 1–6)
Solution injections / polyp (*n*)	3.3 (range: 1–6)	4.1 (range: 1–10)
Polyps re-injected (*n*)	3	2
Injected volume (mL)	30.7 (range: 7–50)	41.1 (range: 20–110)
Polyp lift (min)	63.9 (range: 45–90)	77.4 (range: 40–90) ^a^
Intervention time (min)	75.8 (range: 40–120)	121.8 (range: 45–420)
Polyp lift/intervention time ratio	0.84	0.64
^b^ Time to resorption/intervention time ratio	1	1
**Complications**		
Bleeding during intervention	100%	100%
**Type of bleeding**		
Severe bleeding (%)	0	6.7
Moderate bleeding (%)	6.7	13.4
Mild bleeding (%)	92.4	79.9
Others (%)	0	0
Adverse effects due to the submucosal injection solution	0	0

^a^ Lift of polyps was only recorded up to a maximum time of 90 min. ^b^ We did not observe resorption of injected solution during intervention time in EMR and ESD.
